# Circ_0067835 sponges miR‐324‐5p to induce HMGA1 expression in endometrial carcinoma cells

**DOI:** 10.1111/jcmm.15996

**Published:** 2020-11-10

**Authors:** Yun Liu, Yue Chang, Yixuan Cai

**Affiliations:** ^1^ Department of Obstetrics and Gynecology Beijing Friendship Hospital Affiliated to Capital Medical University Beijing China

**Keywords:** circ_0067835, endometrial carcinoma, HMGA1, miR‐324‐5p

## Abstract

Endometrial cancer is a common gynaecological malignant tumour among women across the world. Circular RNAs (circRNAs) are a novel kind of non‐coding RNAs, and they can play a crucial role in multiple cancers. Nevertheless, the mechanisms of circRNAs in regulating gene expression in endometrial cancer are still unclear. Here, our work sought to focus on the role that circ_0067835 exert in progression and development of endometrial cancer cells. We observed circ_0067835 was markedly elevated in endometrial cancer. Then, changes in endometrial cancer cell (RL95‐2 and HEC‐1B) function were determined after circ_0067835 knockdown. Loss‐of‐functional assays revealed that circ_0067835 down‐regulation significantly repressed RL95‐1 and HEC‐1B cell proliferation, migration and invasion. Bioinformatics analysis, luciferase reporter experiment and RNA pull‐down assay were employed to predict and validate circ_0067835 can bind to miR‐324‐5p. Increase in miR‐324‐5p remarkably depressed the proliferation, migration and invasion of endometrial cancer cells via inhibiting high mobility group A1 (HMGA1). HMGA1 is identified as a vital prognostic biomarker in endometrial cancer. Currently, we reported circ_0067835 was positively correlated with HMGA1 in endometrial cancer. We implied that circ_0067835 was capable of sponging miR‐324‐5p and inducing its downstream target HMGA1 in vitro and in vivo. In conclusion, circ_0067835 can compete with miR‐324‐5p, resulting in HMGA1 up‐regulation, and therefore induce the development of endometrial cancer.

## 1 INTRODUCTION

Endometrial cancer is a common tumour among female in many countries.^1^ Several important factors are displayed to participate in endometrial cancer, including post‐menopausal condition, long‐term use of oestrogen and hypertension.^2^ In spite of great advances have been made in endometrial cancer, there is no effective biomarker for endometrial cancer. Hence, it is important to investigate the detailed mechanisms of endometrial cancer to identify novel treatment strategies.

In recent years, circRNA, microRNAs and lncRNAs are most well‐studied non‐coding RNAs.^3^ CircRNAs have a covalently closed loop with poor ability to code protein.^4^ Many researches have pointed out circRNAs can participate in various processes.^5^, ^6^, ^7^ The biological function of circRNAs in cancers is well explored.^8^ For instance, circ_102171 can induce thyroid cancer development via modulating activation of β‐catenin signalling.^9^ In gastric cancer, circ_100269 is decreased and it can inhibit tumour progression via sponging miR‐630.^10^ In addition, hsa_circ_100395 is able to repress lung cancer progression through regulating miR‐1228 and TCF21.^11^ circ_0067835 has been reported to promote colorectal cancer.^12^ Up to now, the roles of circ_0067835 are not clearly elucidated in endometrial carcinoma development.

microRNAs are famous small non‐coding RNAs that can directly modulate the levels of most mRNAs.^13^, ^14^ It has been suggested that microRNAs are aberrantly expressed in endometrial carcinoma.^15^, ^16^ Recently, circRNAs were proved that they can act as sponges of various microRNAs.^17^ Nevertheless, the roles of circRNAs serving as microRNA sponges are not clearly elucidated in endometrial carcinoma. Emerging evidence has indicated that miR‐324‐5p participates in physiological and pathological processes of various cancers.^18^, ^19^, ^20^ However, its role in endometrial carcinoma remains not clear. In addition, HMGA1 was predicted as a downstream target for miR‐324‐5p in endometrial carcinoma. The function of HMGA1 has been exhibited in multiple cancers, including endometrial carcinoma.^21^, ^22^, ^23^

In this work, it was identified by us that circ_0067835 was highly increased in endometrial carcinoma. In addition, it was indicated that loss of circ_0067835 decreased proliferation, migration and invasion of endometrial carcinoma cells through sponging miR‐324‐5p to inhibit expression of HMGA1.

## 2 MATERIALS AND METHODS

### 2.1 Clinical tissue samples

Endometrial cancer tissues were obtained from 10 female patients with surgical resection at Beijing Friendship Hospital Affiliated to Capital Medical University between May 2016 and May 2018. Meanwhile, 10 normal endometrial specimens were collected from non‐menopausal patient undergoing hysterectomy. No patients were given any chemotherapy or radiation therapy before operation. This work was approved by the Ethics Committee of Beijing Friendship Hospital Affiliated to Capital Medical University officially. Patients provided the written informed consent.

### 2.2 Cell culture

Endometrial carcinoma cells (Ishikawa, HEC1‐B and RL95‐2 cells) and the immortalized endometrial fibroblast cell hESCs were purchased from ATCC. We maintained the cells in RPMI‐1640 medium with 10% FBS, 1% penicillin and 1% streptomycin. Cells were placed in a 5% CO_2_, 37°C incubator.

### 2.3 Transfection assays

Lipofectamine^®^ 3000 (Gibco; Thermo Fisher Scientific) was utilized to conduct the transfection. Short hairpin RNAs (shRNAs) for circ_0067835 knockdown were constructed (Invitrogen). miR‐324‐5p mimics, miR‐324‐5p inhibitors and their corresponding negative controls were obtained from Sangon Biotech. circ_0067835 siRNA and pcDNA3.0‐HMGA1 were obtained from RiboBio.

### 2.4 CCK‐8 assay

CCK‐8 (Beyotime) assay was used. 3 × 10^3^ cells were distributed into 96‐well plates. Meanwhile, 200 µL culture media was used to culture the cells. The assay included two groups, negative control group and circ_0067835 siRNA experimental group. Measurements were carried out at various time‐points. At indicated time‐point, 10 µL CCK‐8 solution was added. We incubate the cells at 37°C for 2 hours. A spectrophotometer (BioTek Instruments) was carried out to test the OD values at 450 nm.

### 2.5 EdU assay

To conduct EdU assay, 50 nmol/L EdU (RiboBio) was used to incubate the cells for 2 hours and cells were fixed using 4% formaldehyde. Afterwards, the cells were treated with 1 mL Cell LightTM EdU Apollo^®^ 488 (RiboBio). The nuclear staining was carried out using DAPI for half an hour. Finally, the fluorescence intensity was tested using an inverted microscope.

### 2.6 Flow cytometry of apoptosis assays

A total of 10^4^ cells were added to a 6‐well plate overnight. After transfected for 48 hours, cells were washed twice using PBS and harvested by trypsin with no EDTA. After the supernatant was discarded, 50 μL 1 × binding solution was added. Afterwards, 5 μL annexin V‐FITC and 5 μL PI were added for 15 minutes. Subsequently, 400 μL 1 × binding buffer was used. Apoptosis was determined using flow cytometry.

### 2.7 Colony formation

Cells were grown into 60‐mm dishes and incubated for 2 weeks. After fixed using methanol for 15 minutes, cells were stained using 0.1% crystal violet for half an hour. We counted the colonies using a light microscope.

### 2.8 Scratch assay

Cells were seeded into 6‐well plates for a whole night. In the middle part of the well, a sterile pipette tip was employed to make a scratch. We measured the migration of cells at 48 hours. A light microscope was utilized, and the migration was examined by a calliper via evaluating scratch distance.

### 2.9 Transwell assay

An 8.0‐µm pore insert in a 24‐well Transwell chamber plate was used to conduct invasion assay. 3 × 10^5^ cells were grown to the upper chamber coated with Matrigel. Next, the lower chamber was added with 500 µL RPMI‐1640 with 10% FBS. Forty‐eight hours later, we stained the cells migrating to the bottom of the filter using 0.1% crystal violet for 10 minutes. Finally, we counted the cells staying on the lower side using a light microscope.

### 2.10 Western blot assay

Cell extracts were carried out using cell lysis reagent. Total protein was quantified by a bicinchoninic acid assay. Thirty microgram protein was loaded on 10% SDS‐PAGE and transferred to PVDF membranes. Then, the membranes were blocked using 10% goat serum for an hour. Afterwards, primary antibodies were used. The primary antibodies included E‐cadherin antibody, N‐cadherin, HMGA1, BAX, Bcl‐2 and GAPDH (1:1000 dilution; Abcam). Next day, incubation with secondary antibodies (1:2000 dilution; Abcam) was followed at 37°C for 1 hour. Finally, the protein bands were visualized using the enhanced chemiluminescence reagent.

### 2.11 qRT‐PCR

TRIzol reagent was carried out to isolate total RNA. A First Strand cDNA Synthesis Kit (Takara) was carried to obtain cDNA. Real‐time quantitative PCR was conducted using a SYBR Premix Ex Taq™ II Kit (Takara) using Bio‐Rad CFX96 System. Gene expression was calculated using $2^{‐\Delta \Delta C_{t}}$. Primers are displayed in Table 1.

**Table 1:** Primers used for real‐time PCR

Genes	Forward (5′‐3′)	Reverse (5′‐3′)
GAPDH	GCACCGTCAAGGCTGAGAAC	TGGTG AAGACGCCAGTGGA
Circ_0067835	GCATCCCATTACTTCAGTTGCC	CAGTAGTCCAGCCCACACAG
miR‐324‐5p	GAGGCCAAGCCCTGGTATG	CGGGCCGATTGATCTCAGC
U6	GCTTCGGCAGCACATATACT	AACGCTTCACGAATTTGCGT
HMGA1	AAGACCCGGAAAACCACCAC	GCCCTCCTCTTCCTCCTTCT

### 2.12 Dual‐luciferase reporter assay

To detect luciferase activity, Dual‐Glo Luciferase Assay System (Promega) was carried out. Lipofectamine^®^ 3000 was employed to transfect cloned circ_0067835/HMGA1 wild‐type 3’UTR or mutant 3’UTR purchased from Shanghai GeneChem with miR‐324‐5p mimics, inhibitors or negative control.

### 2.13 Pull‐down experiment with biotinylated circ_0067835 probe

Briefly, 1 × 10^7^ cells were collected. Circ_0067835 probe (RiboBio) was indicated with C‐1 magnetic beads for 2 hours. Then, cell lysates were incubated with circ_0067835 probe or oligo probe for a whole night. RNA complexes binding with the beads were extracted using RNeasy Mini Kit for real‐time PCR.

### 2.14 Pull‐down experiment with biotinylated microRNA

In brief, cells were transfected using biotinylated microRNA mimics or mutant. Forty‐eight hours later, cells were harvested. Cell lysates were incubated with C‐1 magnetic beads for 3 hours and washed using wash buffer. Bound RNAs were purified by RNeasy Mini Kit.

### 2.15 Xenograft assays in vivo

The animal study was approved by the Animal Experimentation Ethics Committee of Beijing Friendship Hospital Affiliated to Capital Medical University. We made great efforts to reduce the pain of the mice. Twelve 6‐week‐old female nude mice were obtained from Shanghai Animal Laboratory Center. Mice were randomly assigned to the control or shRNA group. HEC‐1B cells infected with NC or shRNA (6 × 10^6^) were subcutaneously injected into the mice. Six mice were used in each group. We isolated the xenograft tumours and weighed them. Our research was under the Guide for the Care and Use of Laboratory Animals of the NIH.

### 2.16 Immunohistochemistry

The specimens were fixed using 10% formaldehyde. We embedded the species in paraffin, sectioned them into 5‐µm thick and deparaffinized. To make antigen retrieval, a 96°C water bath was utilized. Afterwards, the sections were treated with 5 mmol/L citrate buffer and 3% H_2_O_2_. Then, 5% goat serum was used to block sections and treated with a Ki‐67 antibody (1:200 dilution; Abcam). A secondary antibody was used to incubate the sections at 37°C for half an hour.

### 2.17 Statistical analysis

Analysis was carried out using SPSS v.22.0 software. To compare between two groups, Student's *t* test was used. A two‐way ANOVA was used to compare between three or more groups. *P* < .05 was considered to be statistically significant.

## 3 RESULTS

### 3.1 Circ_0067835 was overexpressed in endometrial carcinoma

**Figure 1:**
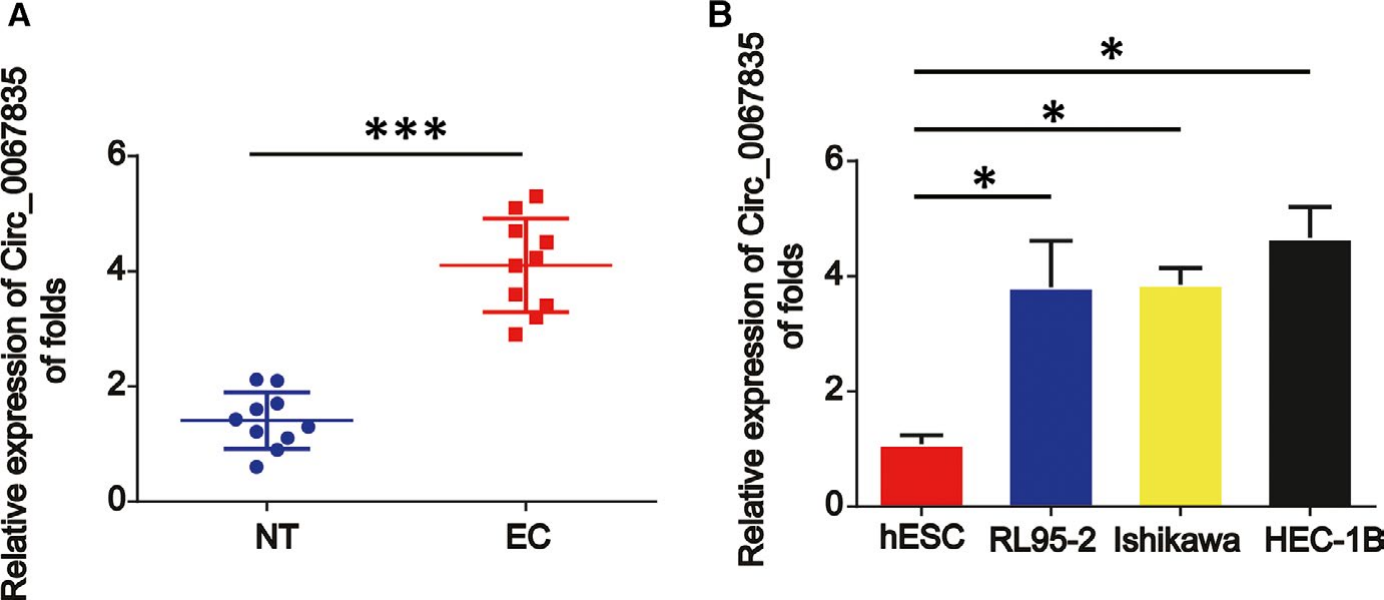
Circ_0067835 was up‐regulated in EC tissues and cells. A, The expression of circ_0067835 was detected by real‐time PCR in endometrial carcinoma and normal endometrial tissues. GAPDH was used as internal control. B, The expression level of circ_0067835 in endometrial carcinoma cells (Ishikawa, HEC‐1B, and RL95‐2) and normal endometrial cells (hESCs). Error bars stand for the mean ± SD of at least triplicate assays. *P < .05, ***P < .001

Circ_0067835 expression was assessed using qRT‐PCR in 10 normal endometrial tissues and 10 endometrial carcinoma tissues. Circ_0067835 was greatly elevated in endometrial carcinoma tissues (Figure 1A). Then, we tested the expression of circ_0067835 in hESCs and endometrial cancer cell lines RL95‐2, Ishikawa and HEC‐1B. As exhibited, the expression of circ_0067835 in endometrial cancer cells was increased than in hESCs (Figure 1B).

### 3.2 Decrease in circ_0067835 reduced proliferation and induced apoptosis of endometrial cancer cells

**Figure 2:**
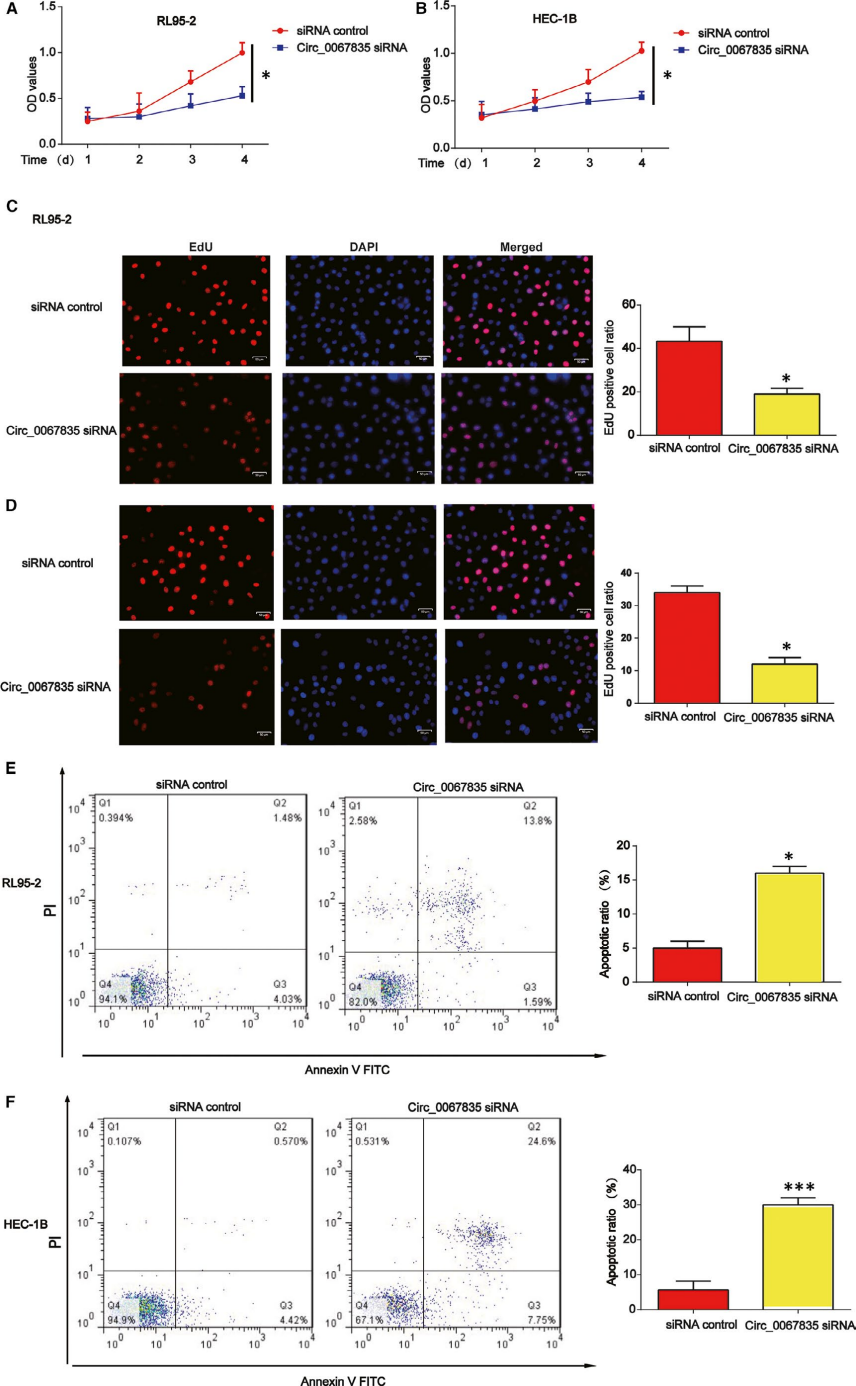
Effects of circ_0067835 siRNA on endometrial carcinoma cell proliferation and apoptosis. A, B, Effects of circ_0067835 siRNA on RL95‐2 and HEC‐1B cell survival. CCK‐8 assay was used to detect cell viability. RL95‐2 and HEC‐1B cells were treated with or without circ_0067835 siRNA. C, D, Effects of circ_0067835 siRNA on RL95‐2 and HEC‐1B cell proliferation. EdU assay was utilized to test cell proliferation. E, F, Effects of circ_0067835 siRNA on RL95‐2 and HEC‐1B cell apoptosis. Flow cytometry assay was carried out to detect cell apoptosis. Error bars stand for the mean ± SD of at least triplicate assays. *P < .05, ***P < .001

Circ_0067835 siRNA was transfected into RL95‐2 and HEC‐1B cells, and we found that circ_0067835 siRNA was able to repress endometrial cancer cell survival in Figure 2A,B. Meanwhile, EdU assay indicated that endometrial cancer cell proliferation was also reduced by circ_0067835 siRNA (Figure 2C,D). Apoptosis assays proved that decrease in circ_0067835 induced cell apoptosis (Figure 2E,F).

### 3.3 Knockdown of circ_0067835 restrained endometrial cancer cell migration and invasion

**Figure 3:**
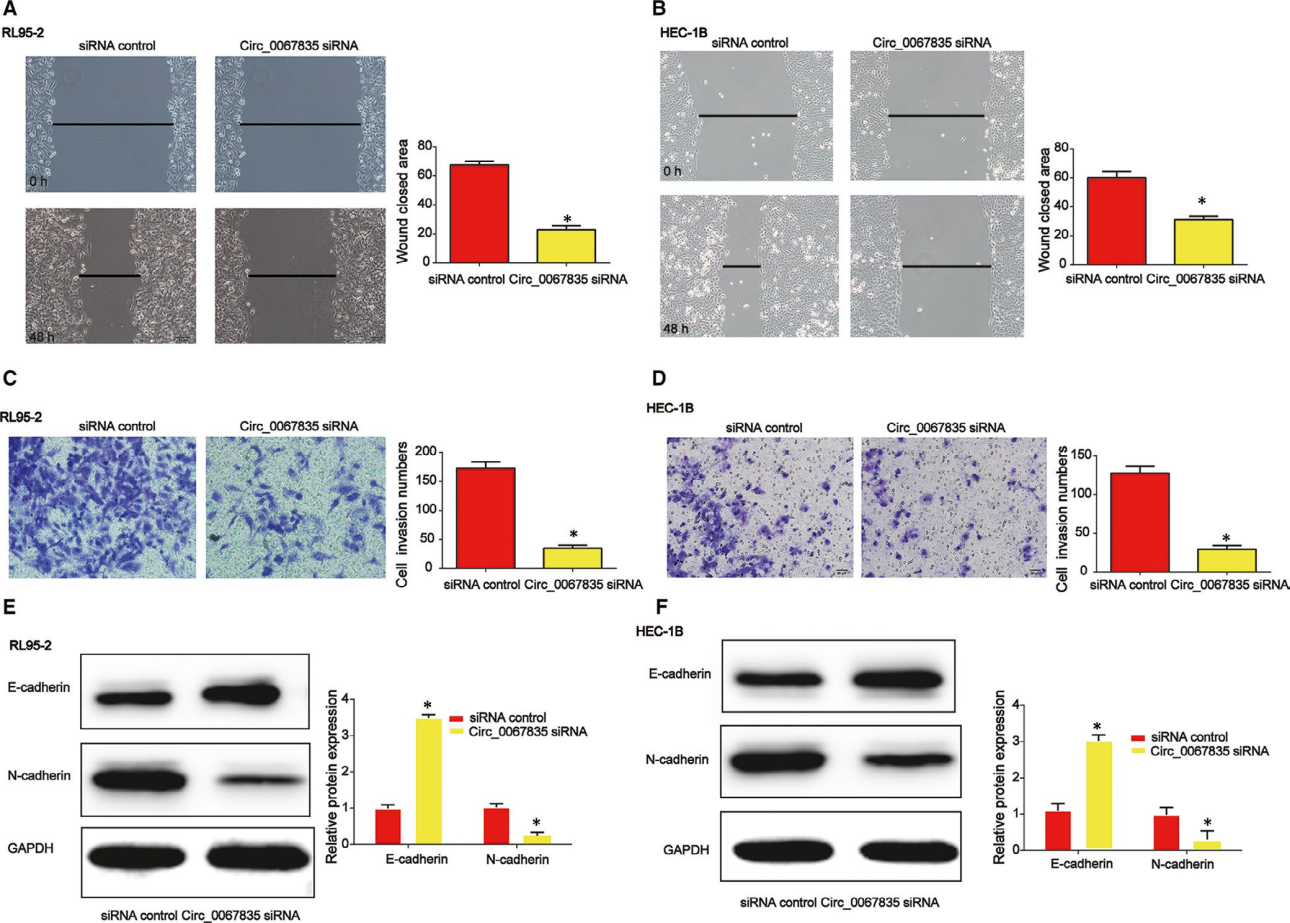
Effects of circ_0067835 siRNA on endometrial carcinoma cell migration and invasion. A, B, Effects of circ_0067835 siRNA on RL95‐2 and HEC‐1B cell migration. Wound‐healing assay was carried out to detect cell migration capacity. C, D, Effects of circ_0067835 siRNA on RL95‐2 and HEC‐1B cell invasion. Transwell invasion assay was employed to detect cell invasion capacity. E, F E‐cadherin and N‐cadherin protein expression level in RL95‐2 and HEC‐1B cells. Error bars stand for the mean ± SD of at least triplicate assays. *P < .05

Through performing scratch assays and Transwell invasion assays, we proved cell migration and invasion were repressed after circ_0067835 was down‐regulated for 48 hours (Figure 3A‐D). In addition, we reported that E‐cadherin protein expression was increased by circ_0067835 siRNA whereas N‐cadherin protein level was suppressed by loss of circ_0067835 as demonstrated in Figure 3E,F.

### 3.4 Circ_0067835 abundantly sponged miR‐324‐5p in endometrial cancer cells

**Figure 4:**
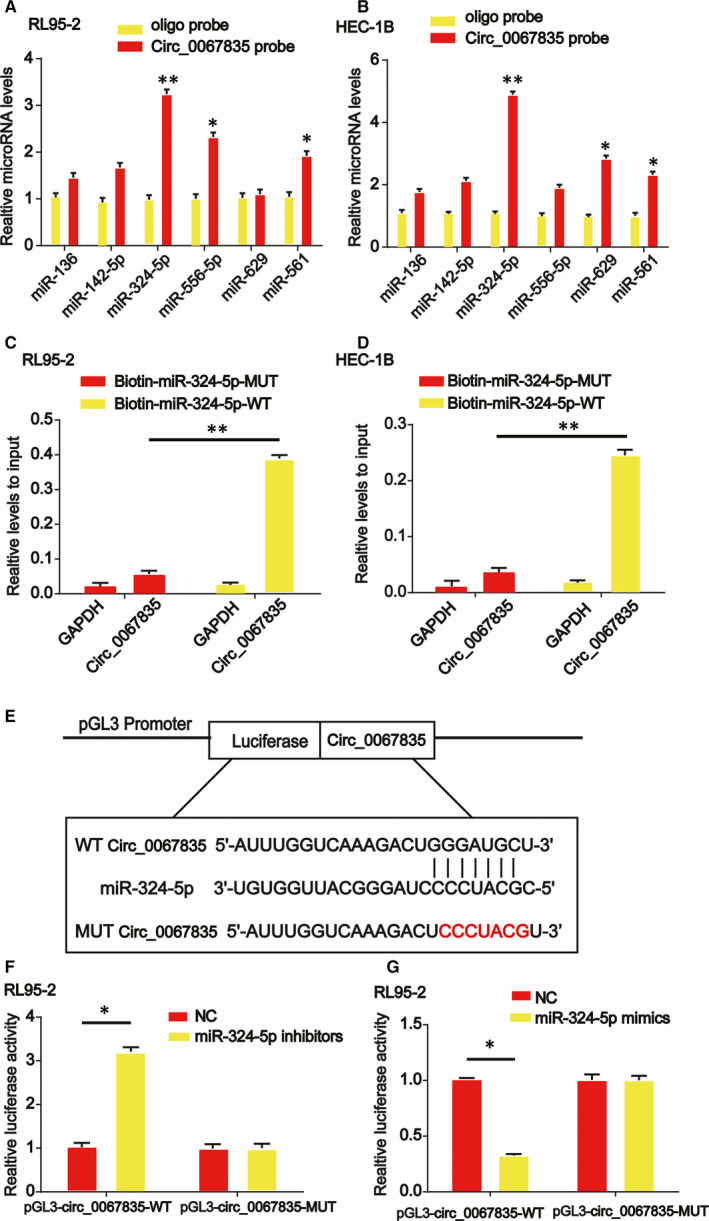
Circ_0067835 sponged miR‐324‐5p in RL95‐2 and HEC‐1B cells. A, B, The relative level of six miRNA candidates in RL95‐2 and HEC‐1B cell lysates was detected by real‐time PCR. Multiple miRNAs were pulled down by circ_0067835, and miR‐324‐5p was pulled down by circ_0067835 in two cell lines. C, D The biotinylated wild‐type miR‐324‐5p (Bio‐miR‐324‐5p‐WT) or its mutant (Bio‐miR‐324‐5p‐MUT) was transfected into RL95‐2 and HEC‐1B cells. After streptavidin capture, circ_0067835 level was quantified by real‐time PCR, and the relative immunoprecipitate (IP)/input ratios were shown. GAPDH was used as negative control. E, The putative binding sites between miR‐324‐5p and circ_0067835 and the mutant sites in circ_0067835‐MUT reporter were displayed. F, Luciferase activity was evaluated in RL95‐2 cells cotransfected with circ_0067835‐WT or circ_0067835‐MUT reporter and miR‐324‐5p inhibitors or its scramble control (NC). G, Luciferase activity was evaluated in HEC‐1B cells cotransfected with circ_0067835‐WT or circ_0067835‐MUT reporter and miR‐324‐5p mimics or its scramble control (NC). Error bars stand for the mean ± SD of at least triplicate assays. *P < .05, **P < .01

In order to find out whether circ_0067835 sponged microRNAs in endometrial cancer cells, a biotin‐labelled circ_0067835 probe was designed. Then, https://circinteractome.nia.nih.gov/ was used to select miR‐324‐5p as the microRNA candidates for circ_0067835. In Figure 4A,B, miR‐324‐5p was most abundantly pulled down by circ_0067835 in vitro. In addition, an increased enrichment of circ_0067835 in the captured fraction of wild‐type miR‐324‐5p was observed (Figure 4C,D). Then, dual‐luciferase reporter gene data suggested that circ_0067835 was capable of sponging miR‐324‐5p. Luciferase reporter plasmids of WT‐circ_0067835 and MUT‐circ_0067835 binding sites were indicated in Figure 4E. Cotransfection of the WT‐circ_0067835 with miR‐324‐5p inhibitors increased the reporter activity in RL95‐2 cells (Figure 4F). In addition, cotransfection of WT‐circ_0067835 with miR‐324‐5p mimics reduced the reporter activity in HEC‐1B cells (Figure 4G).

### 3.5 miR‐324‐5p was decreased in endometrial cancer and restrained cell growth and invasion through targeting HMGA1

**Figure 5:**
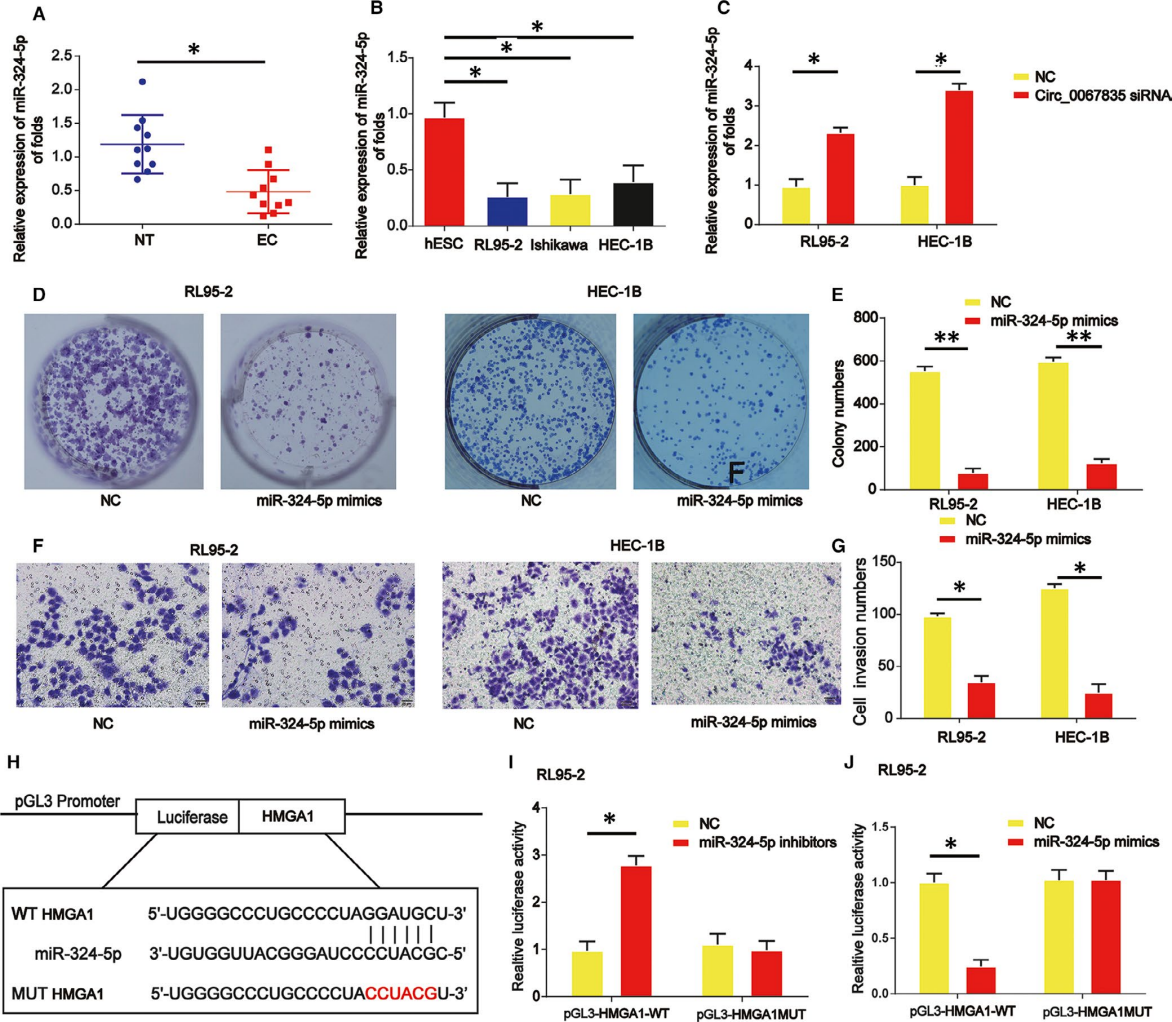
miR‐324‐5p induced enhancement of RL95‐2 and HEC‐1B cell proliferation, migration and invasion via targeting HMGA1. A, The expression of miR‐324‐5p was detected by real‐time PCR in 10 pairs of endometrial carcinoma and normal endometrial tissues. B, The expression level of miR‐324‐5p in endometrial carcinoma cells (Ishikawa, HEC‐1B, and RL95‐2) and normal endometrial cells (hESCs). C, The expression of miR‐324‐5p in RL95‐2 and HEC‐1B cells treated with or without circ_0067835 siRNA. D, E, RL95‐2 and HEC‐1B cell colony formation. F, G, RL95‐2 and HEC‐1B cell invasion capacity. H, The putative binding sites between miR‐324‐5p and HMGA1 and the mutant sites in HMGA1‐MUT reporter were displayed. I, Luciferase activity was evaluated in RL95‐2 cells cotransfected with HMGA1‐WT or HMGA1‐MUT reporter and miR‐324‐5p inhibitors or its scramble control (NC). J, Luciferase activity was evaluated in HEC‐1B cells cotransfected with HMGA1‐WT or HMGA1‐MUT reporter and miR‐324‐5p mimics or its scramble control (NC). Error bars stand for the mean ± SD of at least triplicate assays. *P < .05, **P < .01

We observed miR‐324‐5p was reduced in endometrial cancer tissues and cells as shown in Figure 5A,B. miR‐324‐5p was greatly induced by circ_0067835 siRNA in vitro. (Figure 5C) Then, colony formation assay displayed that miR‐324‐5p mimics greatly repressed cell colony numbers as shown in Figure 5D,E. Additionally, miR‐324‐5p overexpression reduced RL95‐2 and HEC‐1B cell invasion capacity as demonstrated in Figure 5F,G. Luciferase reporter plasmids of WT‐HMGA1 and MUT‐HMGA1 binding sites were manifested in Figure 5H. Cotransfection of WT‐HMGA1 with miR‐324‐5p inhibitors enhanced the reporter activity in RL95‐2 cells (Figure 5I). Cotransfection of WT‐HMGA1 with miR‐324‐5p mimics reduced the reporter activity in HEC‐1B cells (Figure 5J).

### 3.6 A positive correlation between circ_0067835 and HMGA1

**Figure 6:**
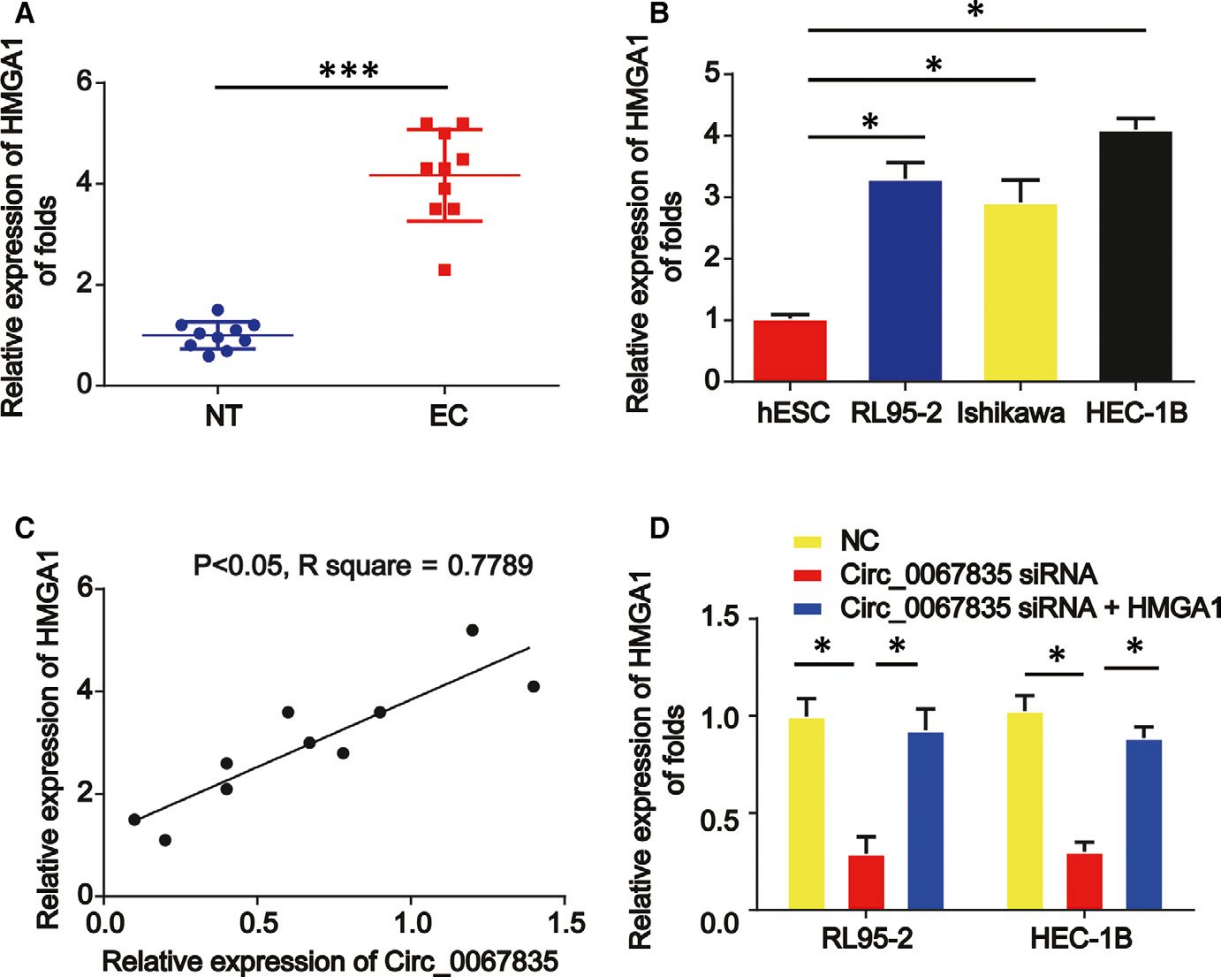
Circ_0067835 positively regulated HMGA1. A, The mRNA expression of HMGA1 in 10 pairs of endometrial carcinoma and normal endometrial tissues. B, The mRNA expression level of HMGA1 in endometrial carcinoma cells (Ishikawa, HEC‐1B, and RL95‐2) and normal endometrial cells (hESCs). C, Correlation analysis of circ_0067835 and HMGA1 mRNA expressions in 10 pairs of endometrial carcinoma and normal endometrial tissues. D, The mRNA expression of HMGA1 in RL95‐2 and HEC‐1B cells treated with circ_0067835 siRNA or circ_0067835 siRNA and HMGA1 overexpression plasmid. Error bars stand for the mean ± SD of at least triplicate assays. *P < .05, ***P < .001

Then, we found HMGA1 was also increased in endometrial cancer tissues and cell lines in Figure 6A,B. A positive correlation between circ_0067835 and HMGA1 in endometrial cancer tissues was analysed using Spearmen's correlation (Figure 6C). It was confirmed that HMGA1 was decreased after knockdown of circ_0067835 in RL95‐2 cells, which was reversed by the increased HMGA1 in endometrial cancer cells (Figure 6D).

### 3.7 Down‐regulated expression of circ_0067835 suppressed the growth of endometrial cancer in vivo

**Figure 7:**
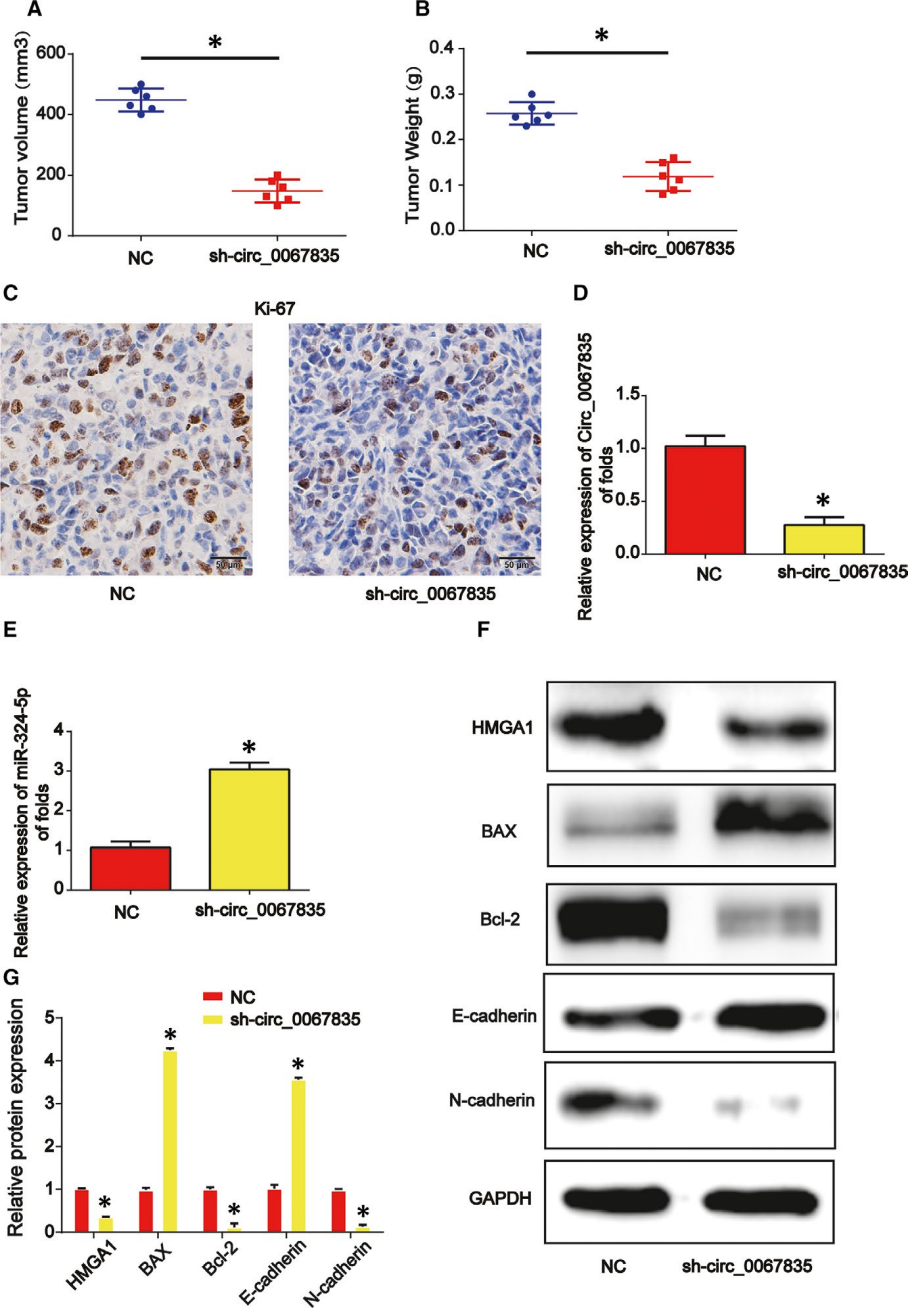
Down‐regulation of circ_0067835 repressed endometrial carcinoma progression through regulating miR‐324‐5p and HMGA1 in vivo. Twelve 8‐wk‐old female BALB/c nude mice were injected with HEC‐1B cells infected with shRNA‐NC (six mice) or circ_0067835 shRNA (six mice). A, Tumour volume. B, Tumour weight. C, IHC staining of Ki‐67 in tumour tissues. D, Expression of circ_0067835 in the tumour tissues from the mice. E, Expression of miR‐324‐5p. F, G, Protein expression of HMGA1, E‐cadherin, N‐cadherin, BAX and Bcl‐2. Three independent experiments were carried out. Error bars stand for the mean ± SD of at least triplicate experiments. *P < .05

Next, we studied the effects of low expression of circ_0067835 on tumour growth in vivo. HEC‐1B cells transfected with circ_0067835 shRNA or control vector were injected into BALB/c nude mice subcutaneously. In Figure 7A,B, the decreased tumour volume and tumour weight were exhibited in circ_0067835 shRNA group. In Figure 7C, immunohistochemical staining implied that the expression of Ki‐67 was inhibited by circ_0067835 shRNA (Figure 6D). Finally, we revealed that circ_0067835 inhibition was able to repress HMGA1 protein expression via sponging miR‐324‐5p (Figure 6E‐G). Meanwhile, apoptosis marker Bax was increased whereas Bcl‐2 expression was decreased by circ_0067835 shRNA in Figure 6F,G. EMT‐associated marker E‐cadherin protein level was induced and N‐cadherin was reduced by loss of circ_0067835 (Figure 6F,G).

## 4 DISCUSSION

It is widely recognized that circRNAs can play crucial functions in regulating genes.^24^, ^25^ Recently, circRNAs are shown to be dysregulated in a variety of cancers.^26^ Therefore, the biological functions of circRNAs are becoming a hot topic. For instance, Circ_BCRC‐3 can repress bladder cancer growth via regulating miR‐182‐5p and p27.^27^ Circ_NEK6 promotes thyroid cancer through sponging miR‐370‐3p and regulating Wnt.^28^ In addition, circRNAs have been implicated in endometrial cancer.^29^, ^30^ For example, circ_PUM1 can contribute to endometrial cancer through targeting miR‐136 and NOTCH3.^31^

CircRNA‐0067835 can regulate liver fibrosis development via acting as miR‐155 sponge and increasing FOXO3a levels.^32^ It has been revealed that circ‐0067835 and miR‐155 are involved in temporal lobe epilepsy.^33^ Currently, we found that circ_0067835 was obviously enhanced in endometrial cancer tissues and cells relative to normal ones. To study the role of circ_0067835 in endometrial cancer, loss of circ_0067835 was designed. RL95‐2 and HEC‐1B cells were transfected with circ_0067835 to evaluate its effects on cell progression. Cells down‐regulating circ_0067835 displayed an obviously reduced proliferation, increased apoptosis and repressed migration and invasion ability. HEC‐1B cells with circ_0067835 shRNA were injected subcutaneously into nude mice. As shown, the tumour volume and tumour weight were remarkably smaller. These data suggested that circ_0067835 exhibited an important role in endometrial cancer. Many researchers have reported that circRNAs can serve as ceRNAs during carcinogenesis and cancer progression. The roles of circRNAs in modulating microRNAs are becoming a focus recently. Currently, miR‐324‐5p was predicted as a target of circ_0067835. miR‐324‐5p can inhibit hepatocellular carcinoma cell progression via post‐transcriptionally decreasing ETS1 and SP1.^34^ miR‐324‐5p can repress glioma cell proliferation through targeting GLI1.^35^ The close interaction between miR‐324‐5p and circ_0067835 was proved in endometrial carcinoma cells. Additionally, increase in miR‐324‐5p suppressed endometrial carcinoma cell colony formation and invasion capacity.

HMGA1 is an architectural transcription factor, and it is involved in a number of tumour processes. For instance, HMGA1 can promote cervical cancer tumour growth via targeting miR‐221/222.^36^ microRNA‐758 reduces the osteosarcoma malignant phenotypes through targeting HMGA1 and Wnt signalling.^37^ miR‐4458 can restrain migration and EMT through targeting HMGA1 in lung cancer.^38^ In addition, in endometrial cancer, increase in HMGA1 can function as an effective prognostic factor.^23^ Based on these data, we examined HMGA1 expression in endometrial carcinoma tissues and cells. A positive correlation between HMGA1 and circ_0067835 was indicated. Loss of circ_0067835 repressed HMGA1 expression greatly in RL95‐2 and HEC‐1B cells. miR‐324‐5p could directly interact with HMGA1 through binding with its 3’UTR sites. It has been shown that circRNAs act as miRNA sponges to modulate the expression of tumour regulatory genes through circRNA‐miRNA‐mRNA axis. In our study, we displayed that circ_0067835 functions as a sponge absorbing miR‐324‐5p to modulate HMGA1 expression in endometrial carcinoma. Moreover, some circRNAs can gather in the nucleus and bind to the linear transcripts of their parental genes to regulate mRNA translation.^39^ In addition, some circRNAs are translated into proteins to exert their crucial biological function.^40^ Further functional mechanism of circ_0067835 in endometrial carcinoma should be focused on and additional future research will be needed to address this. In addition, more endometrial carcinoma clinical samples and cells can be included. Some other certain circRNAs and the detailed mechanism in endometrial carcinoma should be well explored. To conclude, we displayed circ_0067835 was increased in endometrial carcinoma and it sponged miR‐324‐5p to induce HMGA1. Loss of circ_0067835 could significantly inhibit progression of endometrial carcinoma cells via targeting miR‐324‐5p/HMGA1 axis. Our data pointed out a novel therapeutic target for endometrial carcinoma.

## CONFLICT OF INTEREST

The authors confirm that there are no conflicts of interest.

## AUTHOR CONTRIBUTIONS

**Yun Liu:** Conceptualization (lead); Project administration (lead); Resources (lead); Writing‐review & editing (lead). **Yue Chang:** Formal analysis (equal); Methodology (equal); Software (equal). **Yixuan Cai:** Data curation (equal); Investigation (equal); Validation (equal); Visualization (equal).

## Data Availability

The data that support the findings of this study are available from the corresponding author upon reasonable request.

## References

[1] Jemal A , Siegel R , Ward E , Murray T , Xu J , Thun MJ . Cancer statistics, 2007. CA Cancer J Clin. 2007;57:43‐66. 17237035 10.3322/canjclin.57.1.43

[2] Fong P , Meng LR . Effect of mTOR inhibitors in nude mice with endometrial carcinoma and variable PTEN expression status. Med Sci Monit Basic Res. 2014;20:146‐152. 25266877 10.12659/MSMBR.892514 PMC4189716

[3] Smolle E , Haybaeck J . Non‐coding RNAs and lipid metabolism. Int J Mol Sci. 2014;15:13494‐13513. 25093715 10.3390/ijms150813494 PMC4159807

[4] Memczak S , Jens M , Elefsinioti A , et al. Circular RNAs are a large class of animal RNAs with regulatory potency. Nature. 2013;495:333‐338. 23446348 10.1038/nature11928

[5] Xu N , Chen S , Liu Y , et al. Profiles and bioinformatics analysis of differentially expressed circrnas in taxol‐resistant non‐small cell lung cancer cells. Cell Physiol Biochem. 2018;48:2046‐2060. 30099455 10.1159/000492543

[6] Patop IL , Kadener S . circRNAs in cancer. Curr Opin Genet Dev. 2018;48:121‐127. 29245064 10.1016/j.gde.2017.11.007 PMC5877416

[7] Li X , Yang L , Chen LL . The biogenesis, functions, and challenges of circular RNAs. Mol Cell. 2018;71:428‐442. 30057200 10.1016/j.molcel.2018.06.034

[8] Kristensen LS , Hansen TB , Veno MT , Kjems J . Circular RNAs in cancer: opportunities and challenges in the field. Oncogene. 2018;37:555‐565. 28991235 10.1038/onc.2017.361 PMC5799710

[9] Bi W , Huang J , Nie C , et al. CircRNA circRNA_102171 promotes papillary thyroid cancer progression through modulating CTNNBIP1‐dependent activation of beta‐catenin pathway. J Exp Clin Cancer Res. 2018;37:275. 30424816 10.1186/s13046-018-0936-7 PMC6234664

[10] Zhang Y , Liu H , Li W , et al. CircRNA_100269 is downregulated in gastric cancer and suppresses tumor cell growth by targeting miR‐630. Aging. 2017;9:1585‐1594. 28657541 10.18632/aging.101254 PMC5509457

[11] Chen D , Ma W , Ke Z , Xie F . CircRNA hsa_circ_100395 regulates miR‐1228/TCF21 pathway to inhibit lung cancer progression. Cell Cycle. 2018;17:2080‐2090. 30176158 10.1080/15384101.2018.1515553 PMC6224268

[12] Feng W , Gong H , Wang Y , et al. circIFT80 functions as a ceRNA of miR‐1236‐3p to promote colorectal cancer progression. Mol Ther Nucleic Acids. 2019;18:375‐387. 31648103 10.1016/j.omtn.2019.08.024 PMC6819894

[13] Mohr AM , Mott JL . Overview of microRNA biology. Semin Liver Dis. 2015;35:3‐11. 25632930 10.1055/s-0034-1397344 PMC4797991

[14] Bhaskaran M , Mohan M . MicroRNAs: history, biogenesis, and their evolving role in animal development and disease. Vet Pathol. 2014;51:759‐774. 24045890 10.1177/0300985813502820 PMC4013251

[15] Ferlita A , Battaglia R , Andronico F , et al. Non‐coding RNAs in endometrial physiopathology. Int J Mol Sci. 2018;19:2120. 10.3390/ijms19072120 PMC6073439 30037059

[16] Umene K , Banno K , Kisu I , et al. New candidate therapeutic agents for endometrial cancer: potential for clinical practice (review). Oncol Rep. 2013;29:855‐860. 23291663 10.3892/or.2013.2221 PMC3597537

[17] Hansen TB , Jensen TI , Clausen BH , et al. Natural RNA circles function as efficient microRNA sponges. Nature. 2013;495:384‐388. 23446346 10.1038/nature11993

[18] Tang B , Xu A , Xu J , et al. MicroRNA‐324‐5p regulates stemness, pathogenesis and sensitivity to bortezomib in multiple myeloma cells by targeting hedgehog signaling. Int J Cancer. 2018;142:109‐120. 28905994 10.1002/ijc.31041

[19] Zhi T , Yu T , Pan M , et al. EZH2 alteration driven by microRNA‐524‐5p and microRNA‐324‐5p promotes cell proliferation and temozolomide resistance in glioma. Oncotarget. 2017;8:96239‐96248. 29221202 10.18632/oncotarget.21996 PMC5707096

[20] Sun LN , Xing C , Zhi Z , et al. Dicer suppresses cytoskeleton remodeling and tumorigenesis of colorectal epithelium by miR‐324‐5p mediated suppression of HMGXB3 and WASF‐2. Oncotarget. 2017;8:55776‐55789. 28915552 10.18632/oncotarget.18218 PMC5593523

[21] Sumter TF , Xian L , Huso T , et al. The High Mobility Group A1 (HMGA1) transcriptome in cancer and development. Curr Mol Med. 2016;16:353‐393. 26980699 10.2174/1566524016666160316152147 PMC5408585

[22] Wang Y , Hu L , Zheng Y , Guo L . HMGA1 in cancer: cancer classification by location. J Cell Mol Med. 2019;23:2293‐2302. 30614613 10.1111/jcmm.14082 PMC6433663

[23] Palumbo Junior A , de Sousa VPL , Esposito F , et al. Overexpression of HMGA1 figures as a potential prognostic factor in endometrioid endometrial carcinoma (EEC). Genes. 2019;10:372. 10.3390/genes10050372 PMC6562754 31096664

[24] Conn SJ , Pillman KA , Toubia J , et al. The RNA binding protein quaking regulates formation of circRNAs. Cell. 2015;160:1125‐1134. 25768908 10.1016/j.cell.2015.02.014

[25] Bachmayr‐Heyda A , Reiner AT , Auer K , et al. Correlation of circular RNA abundance with proliferation–exemplified with colorectal and ovarian cancer, idiopathic lung fibrosis, and normal human tissues. Sci Rep. 2015;5:8057. 25624062 10.1038/srep08057 PMC4306919

[26] Meng S , Zhou H , Feng Z , et al. CircRNA: functions and properties of a novel potential biomarker for cancer. Mol Cancer. 2017;16:94. 28535767 10.1186/s12943-017-0663-2 PMC5440908

[27] Xie F , Li Y , Wang M , et al. Circular RNA BCRC‐3 suppresses bladder cancer proliferation through miR‐182‐5p/p27 axis. Mol Cancer. 2018;17:144. 30285878 10.1186/s12943-018-0892-z PMC6169039

[28] Chen F , Feng Z , Zhu J , et al. Emerging roles of circRNA_NEK6 targeting miR‐370‐3p in the proliferation and invasion of thyroid cancer via Wnt signaling pathway. Cancer Biol Ther. 2018;19:1139‐1152. 30207869 10.1080/15384047.2018.1480888 PMC6301817

[29] Chen BJ , Byrne FL , Takenaka K , et al. Analysis of the circular RNA transcriptome in endometrial cancer. Oncotarget. 2018;9:5786‐5796. 29464034 10.18632/oncotarget.23534 PMC5814174

[30] Liu KS , Pan F , Mao XD , Liu C , Chen YJ . Biological functions of circular RNAs and their roles in occurrence of reproduction and gynecological diseases. Am J Transl Res. 2019;11:1‐15. 30787966 PMC6357300

[31] Zong ZH , Liu Y , Chen S , Zhao Y . Circ_PUM1 promotes the development of endometrial cancer by targeting the miR‐136/NOTCH3 pathway. J Cell Mol Med. 2020;24:4127‐4135. 32073729 10.1111/jcmm.15069 PMC7171399

[32] Zhu L , Ren T , Zhu Z , et al. Thymosin‐beta4 mediates hepatic stellate cell activation by interfering with CircRNA‐0067835/miR‐155/FoxO3 signaling pathway. Cell Physiol Biochem. 2018;51:1389‐1398. 30481761 10.1159/000495556

[33] Gong GH , An FM , Wang Y , Bian M , Wang D , Wei CX . Comprehensive circular RNA profiling reveals the regulatory role of the CircRNA‐0067835/miR‐155 pathway in temporal lobe epilepsy. Cell Physiol Biochem. 2018;51:1399‐1409. 30485839 10.1159/000495589

[34] Cao L , Xie B , Yang X , et al. MiR‐324‐5p suppresses hepatocellular carcinoma cell invasion by counteracting ECM degradation through post‐transcriptionally downregulating ETS1 and SP1. PLoS One. 2015;10:e0133074. 26177288 10.1371/journal.pone.0133074 PMC4503725

[35] Xu HS , Zong HL , Shang M , et al. MiR‐324‐5p inhibits proliferation of glioma by target regulation of GLI1. Eur Rev Med Pharmacol Sci. 2014;18:828‐832. 24706306

[36] Fu F , Wang T , Wu Z , et al. HMGA1 exacerbates tumor growth through regulating the cell cycle and accelerates migration/invasion via targeting miR‐221/222 in cervical cancer. Cell Death Dis. 2018;9:594. 29789601 10.1038/s41419-018-0683-x PMC5964147

[37] Ren J , Yang M , Xu F , Chen J . microRNA‐758 inhibits the malignant phenotype of osteosarcoma cells by directly targeting HMGA1 and deactivating the Wnt/beta‐catenin pathway. Am J Cancer Res. 2019;9:36‐52. 30755810 PMC6356927

[38] Ma Y , Li X , Chen S , Du B , Li Y . MicroRNA‐4458 suppresses migration and epithelial‐mesenchymal transition via targeting HMGA1 in non‐small‐cell lung cancer cells. Cancer Manage Res. 2019;11:637‐649. 10.2147/CMAR.S185117 PMC6331073 30666160

[39] Pamudurti NR , Bartok O , Jens M , et al. Translation of CircRNAs. Mol Cell. 2017;66(1):9‐21.e7. 28344080 10.1016/j.molcel.2017.02.021 PMC5387669

[40] Du WW , Zhang C , Yang W , Yong T , Awan FM , Yang BB . Identifying and characterizing circRNA‐protein interaction. Theranostics. 2017;7:4183‐4191. 29158818 10.7150/thno.21299 PMC5695005

